# Observing Cusp High-Altitude Reconnection and Electrodynamics: The TRACERS Student Rocket

**DOI:** 10.1007/s11214-025-01192-4

**Published:** 2025-07-07

**Authors:** Brendan N. Powers, Connor A. Feltman, Allison N. Jaynes, Aidan T. Moore, Tamar Ervin, Kristie LLera, Olivia L. Jones, Brandon L. Burkholder, Jason A. Homann, Arissa S. Khan, David H. Vandercoy-Daniels, Craig A. Kletzing, David M. Miles, John W. Bonnell, Jasper S. Halekas, Stephen A. Fuselier, George B. Hospodarsky, Scott R. Bounds

**Affiliations:** 1 Department of Physics and Astronomy, University of Iowa, Iowa City, IA USA; 2 Department of Physics, University of California, Berkeley, CA USA; 3 Space Sciences Laboratory, University of California, Berkeley, CA USA; 4 Space Science Division, Southwest Research Institute, San Antonio, TX USA; 5 Goddard Planetary Heliophysics Institute, University of Maryland Baltimore County, Baltimore, MD USA; 6 NASA Goddard Space Flight Center, Greenbelt, MD USA; 7 Department of Physics, University of Texas at San Antonio, San Antonio, TX USA

**Keywords:** TRACERS, Cusp, Student, Sounding rocket

## Abstract

Observing Cusp High-altitude Reconnection and Electrodynamics (OCHRE) is a student/early career researcher (ECR) focused sounding rocket that will fly as a compliment to the TRACERS satellites. OCHRE will utilize the deep institutional knowledge of the TRACERS science team to educate and mentor a team of graduate students and ECRs to serve as instrument leads, project manager, and primary investigator. Aiming for a near conjunction with, and at an apogee above, TRACERS in the northern polar cusp, OCHRE will answer some remaining questions from the TRICE-II sounding rockets using TRACERS to contextualize observations in the larger-scale polar cusp dynamics.

## Background

Observing Cusp High-altitude Reconnection and Electrodynamics, OCHRE is a sounding rocket that will compliment the TRACERS (Miles et al. 2025a) twin satellites during their nominal mission. Currently scheduled for the Winter 2026 campaign out of Andøya, Norway, OCHRE will launch on a Black Brandt XII-A configuration with a planned apogee of 1050–1220 km (nominal apogee ∼1150 km $\pm 2 \sigma $) km in the northern polar cusp. With OCHRE observations at approximately twice the TRACERS altitude, and a maximum temporal separation of ∼45 minutes, OCHRE and TRACERS will form an unprecedented three spacecraft view of cusp dynamics. This arrangement is well suited to resolving a variety of cusp dynamics, addressing open questions from the earlier TRICE-II sounding rocket mission (Trattner et al. 2025), and supporting the broader TRACERS science goals.

Additionally, OCHRE is unique in its ‘student forward’ mission concept. Graduate students, post doctoral, and early career researchers, overseeing a team of undergraduates, serve as primary investigator, project manager, and instrument leads. They have decision making power over instrumentation, design choices, and mission critical decisions. While working with Wallops Flight Facility (WFF) Sounding Rocket Program Office (SRPO) and NASA Sounding Rocket Operations Contract (NSROC) engineers, the team also develops cross-discipline communication skills. The student forward model emphasizes learning opportunities for the student/ECR team through mentorship from the TRACERS instrument leads and other members of the science team to maximize the chance of mission success. The following sections will outline the unique team structure of OCHRE, the mission science goals, and instrumentation.

### The ‘Student Forward’ Approach

The OCHRE mission; instrument design decisions, interfacing with WFF and NSROC engineers, and calling the launch are spearheaded by graduate students and post-docs. This student forward concept represents a ground-breaking mission format that contributes directly to the educational and training goals of the TRACERS satellite mission. In answering questions about the altitude-resolved nature of the cusp that TRACERS alone cannot determine, Sect. 2, OCHRE also contributes to the science output of the TRACERS mission and the goals of NASA’s Heliophysics Division.

From the beginning, emphasis has been placed on the fact that the students/ECRs will make decisions, keep schedules, and communicate with NASA officials under their own agency. This pedagogical decision was made to maximize student agency over the mission and provide an opportunity for growth beyond what is currently possible within the context of a traditional mission archetype. The students and ECRs involved in the project get experience in leadership roles far ahead of what is typically accessible to them while a much deeper bench of established researchers at partner institutions are available to supervise individual instrument PIs and help support a successful mission more broadly. In this way, OCHRE provides a unique opportunity for training the next generation of space physics researchers and experimentalists.

The OCHRE team comprises undergraduates, graduate students, and ECR’s from The University of Iowa, the Space Sciences Laboratory at the University of California at Berkeley, Southwest Research Institute and NASA/Goddard Space Flight Center. The OCHRE team tree can be found in Appendix A, Fig. 12, and provides detail on the team roles and structure. Student leads supervise flight fabrication, build, and testing of all instruments, while interfacing directly with WFF SRPO mission manager and respective NSROC flight teams. The significant flight heritage of instruments, as well as TRACERS development work, have enabled this mission concept and greatly lowers the barrier to entry for the team on the hardware fabrication, assembly, and test. Details on the instrumentation are found in Sect. 3.

Through instrument delivery and integration, the student PI will continue managing the science team in order to make launch decisions against a set of pre-determined launch criteria. After launch, the team will produce data files from the flight and hold science meetings to discuss results. Most of the involved graduate students are in their second or third year of graduate school; with the flight occurring within the next year or so, this schedule is well-positioned so that these students can complete a full thesis centered around their experience with the OCHRE mission.

## Science Goals

The primary science objective of OCHRE is to utilize a near conjunction with TRACERS to characterize ion and electron distribution profiles and reconnection signatures in the northern polar cusp. With the chosen instrument suite, mimicking the TRACERS instrumentation, OCHRE is well equipped to measure evidence of dayside reconnection processes across the cusp. This includes ion and electron energy dispersions, associated electromagnetic wave modes generated from reconnection, magnetic field convection and associated electric field, and cusp ion outflow structuring.

The planned apogee of 1050–1220 km (nominal apogee ∼1150 km $\pm 2 \sigma $) is approximately twice the TRACERS altitude at 590 km. With this planned trajectory, OCHRE will measure the altitude profile of cusp structures and reconnection signatures near the TRACERS pass. At a slower ground speed, approximately ∼1 km/s for OCHRE vs. ∼7 km/s for TRACERS, OCHRE produces higher spatial resolution data that will allow for study of electron signatures associated with reconnection beyond what TRACERS alone is capable of. The slower ground speed also enables the use of interferometric electric field measurements on the OCHRE payload that will help discriminate Doppler-shifted spatial structures from temporal fluctuations in the plasma frame. Such discrimination will be beneficial for TRACERS, and is key for identifying the electromagnetic wave modes associated with ion acceleration and heating.

**Fig. 1:**
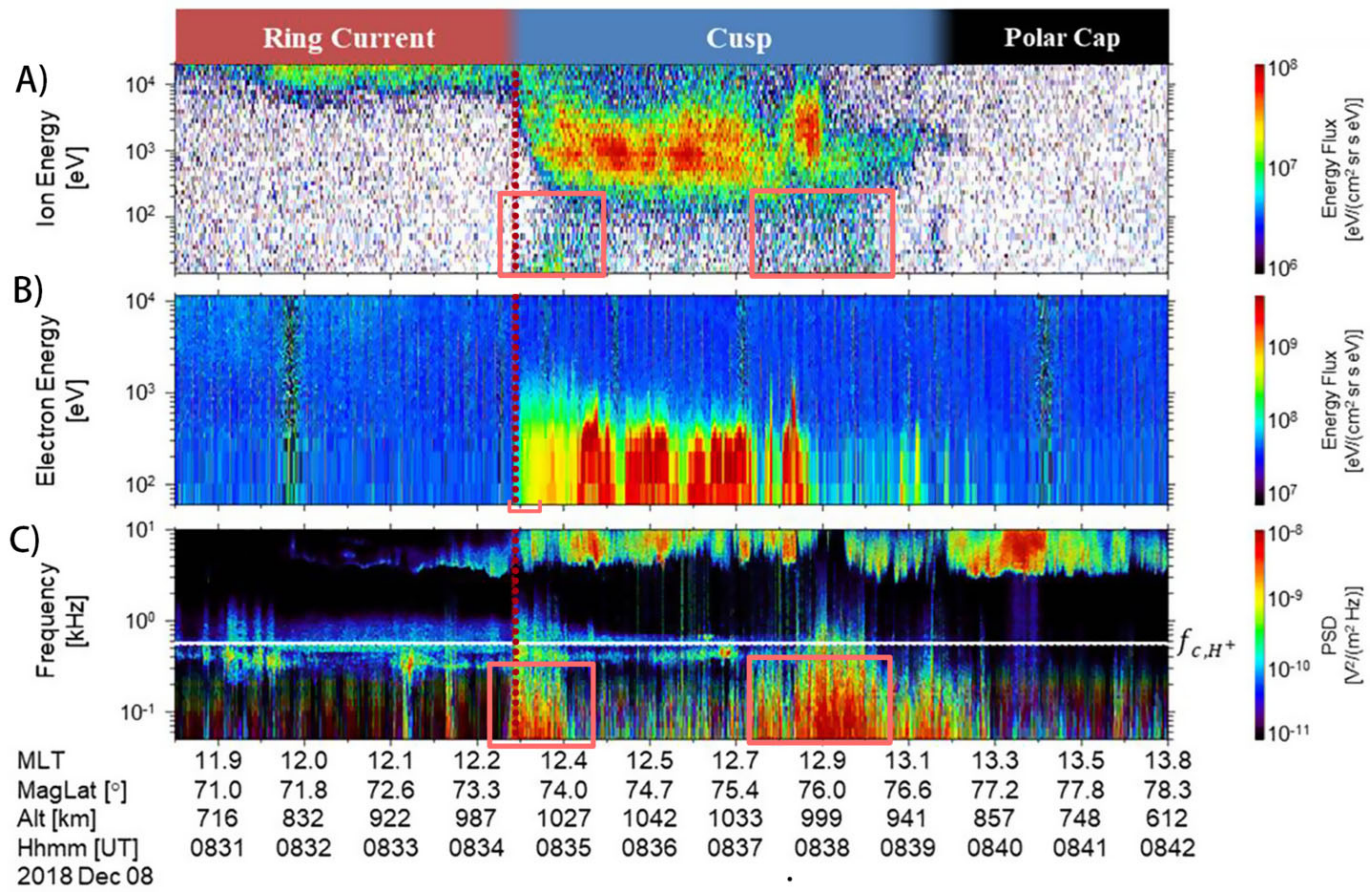
Observations from the TRICE-II High Flyer; A) omni-directional ion energy flux, B) omni-directional electron energy flux, C) electric field Power Spectral Density. The first box highlights the observation of mirroring magnetospheric particles, and the coincident broad band wave features, see Sect. 2.1. The second box highlights the onset of ion outflow, and again the coincident broad band wave features, see Sect. 2.2. The red dotted line marks the open/closed field line boundary (OCB). Adapted from Sawyer et al. (2021)

With the quantity of data available from three point observations of the low altitude cusp with both a temporal and altitudinal separation, OCHRE and TRACERS together will also be able to explore a variety of open questions in the cusp region. Particularly, OCHRE focuses on open questions resulting from the TRICE-II sounding rockets that the increased spatial and temporal coverage from OCHRE and TRACERS will be particularly well suited to answer. A summary of the relevant TRICE-II observations are shown in Fig. 1 and further details can be found in Trattner et al. (2025).

### SQ1: To What Extent do Wave-Particle Interactions with BBELF Waves Dictate Ion Pitch Angle Distributions in the Cusp?

A variety of particle populations inhabit the cusp region, including precipitating shocked solar wind particles and precipitating magnetospheric ions (both populations ∼200 eV for electrons, a few keV for ions), and cold (≤ 10 eV) ionospheric ions. The TRICE-II high flier observed a warm (tens of eV) ion population, concentrated on the poleward edge of the cusp, coincident with so called broad band extremely low frequency (BBELF) waves (Sawyer et al. 2021), see Fig. 1A and 1C. Due to partial deployment failure of the ion instrument on the TRICE-II high flyer, pitch angle measurements above ∼135^∘^ were lost, and the directional flow of these particles could not be confirmed. The TRICE-2 low flyer did not observe this population, while MMS did observe a similar energy population at the magnetosheath during a near conjunction. This led Sawyer et al. (2021) to identify this population as plasma cloak ions (Chappell et al. 2008).

Issues exist with this interpretation. Sawyer et al. (2021) invokes parallel ion heating by the coincident BBELF waves to explain the low mirror point of these particles. Parallel ion heating is not currently understood as a signature of BBELF wave-particle interactions; Rather, BBELF observations are almost always associated with transverse ion heating, the formation of ion conics, and subsequent ion outflow (André et al. 1994; Yau and André 1997; André et al. 1998; Shen et al. 2018). Also, unless the observed cloak particles were characterized as being significantly less field aligned than expected, than it is unclear why parallel heating is necessary to explain this observation.

These broadband wave features are common observations throughout the high latitude ionosphere (André and Yau 1997; André et al. 1998; Su et al. 2001). Typically observed across a broad range of frequencies ($\le 4$ kHz), these broadband features have been previously identified as Doppler shifted dispersive Alfvén waves (Chaston et al. 2004), electrostatic ion cyclotron waves (Kintner et al. 1996), electromagnetic ion cyclotron waves (Erlandson et al. 1994), slow ion acoustic waves (Kintner et al. 1996), or some combination of the above wave modes (Kintner et al. 1996; André et al. 1998; Shen et al. 2018).

One alternate explanation of this warm ion observation is that these ions were ambient ionospheric ions that were heated by the BBELF wave signatures, rather than the precipitating magnetospheric ions as proposed by Sawyer et al. (2021). For these heated ions, the mirror force would quickly overcome gravity and force them to escape along magnetic field lines; meaning they would typically not be observable at TRICE-II altitudes. A scenario proposed by Gorney et al. (1985), has been inferred to explain similar observations of transversely accelerated ions (TAIs) by the Freja, FAST, (∼ 4000 km) (Marklund 2009), and CASSIOPE satellites(∼400–700 km) (Shen et al. 2018). The so called ‘Pressure Cooker’ scenarios invokes a downward parallel electric field to hold ions in the BBELF heating region for an extended period of time.

**Fig. 2:**
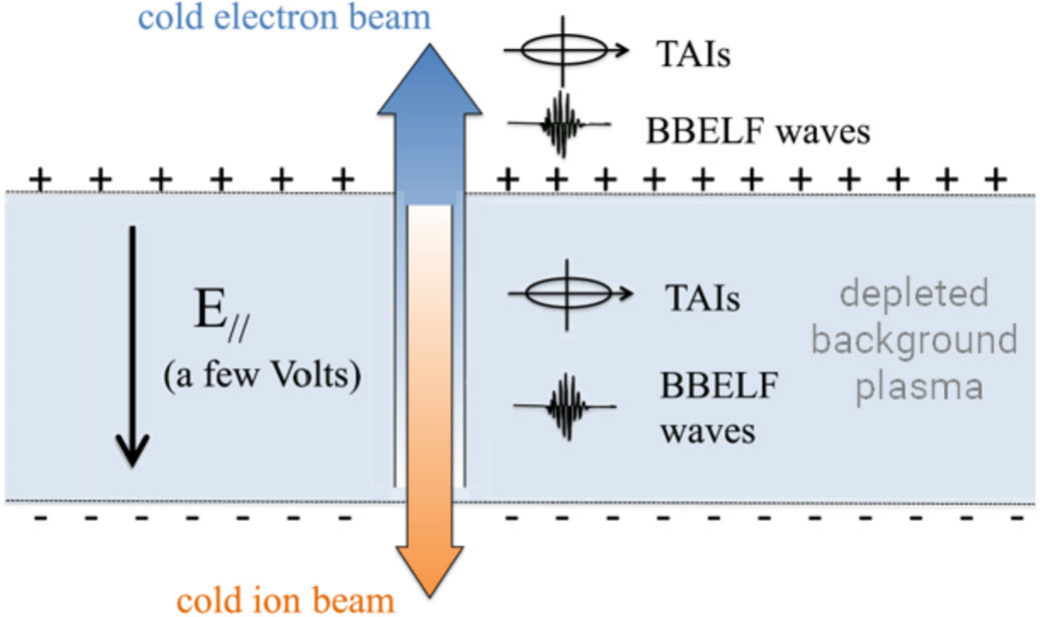
A diagram of a ‘Pressure Cooker’ scenario. The BBELF waves act to transversely heat ambient ions, while the very cold ions get forced to lower altitudes. These transversely accelerated ions (TAIs) eventually escape the parallel electric field where they would be observed as ion upflow. Adapted from Shen et al. (2018)

In this situation, TRICE-II could have flown through the BBELF heating region of the pressure cooker; therefore, the observed population would presumably be upflowing/transversely heated ionospheric ions that had not yet been heated enough to break out of the parallel electric field. Figure 2 details several proposed bidirectional field aligned signatures of these types of scenarios. Together, OCHRE and TRACERS are well instrumented and well situated to observe these cross directional particle beams, quantify the occurrence regions of these broadband wave features, explore details of these pressure cooker regions and help further shed light on BBELF ion pitch angle structuring.

### SQ2: Is There a Prevailing Spatial Structure to Ion Outflows in the Cusp?

**Fig. 3:**
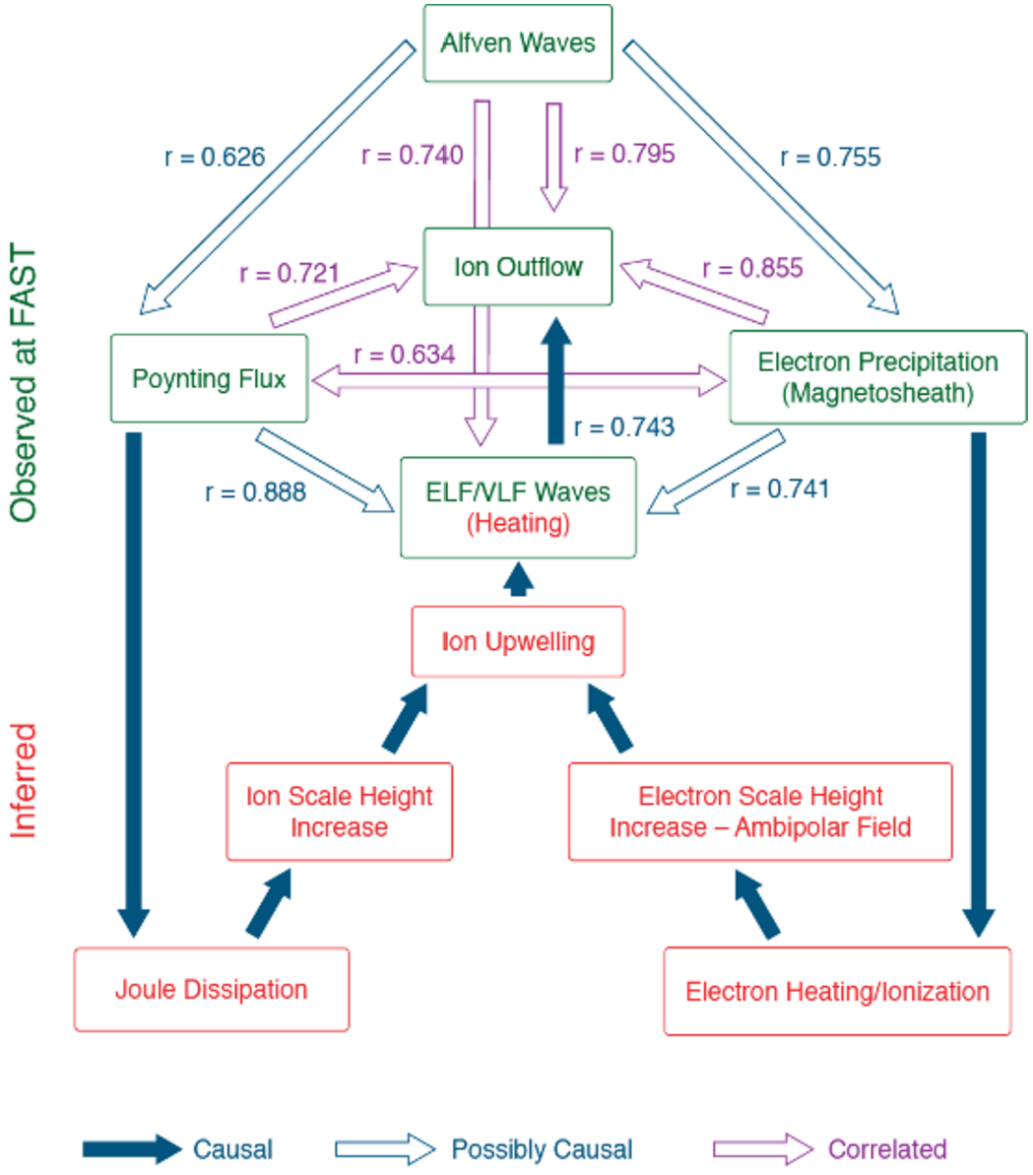
The common pathways of cusp outflows, from direct energy deposition from the solar wind. As observed from FAST satellite observations. Here r is the calculated correlation coefficient between related observations. Provided by R. J. Strangeway, adapted figure from Strangeway et al. (2025). This figure includes the correlation coefficients for the Alfvén-wave related scaling laws presented by Brambles et al. (2011)

Cusp ion outflow is mediated by the input of energy across the magnetopause (Strangeway et al. 2000; Lennartsson et al. 2004; Strangeway et al. 2005). Figure 3 presents the two main pathways of energy deposition into the cusp from electromagnetic energy on the left hand side and particle (thermal) energy on the right hand side. Towards the poleward edge of the cusp, TRICE-II observed warm ion upflow of tens of eV up to ∼100 eV, again coincident with BBELF wave features, see Fig. 1A between 8:37:00 and 8:38:30 UTC. In context of Fig. 3 it can be inferred that TRICE-II observed the initial ion upwelling (into the ELF/VLF heating region) and onset of ion heating with the ELF/VLF wave signatures. As the electron precipitation was not coincident with this observation, the initial ion upwelling most likely was initiated by the raising of the ion scale height as a result of Joule heating from field aligned currents (FACs), left hand side/dark blue arrows in Fig. 3. TRICE-II also launched into B_*y*_ dominant IMF, which causes stronger Region 1 FACs to subsume the usually distinct cusp FACs (Strangeway et al. 2000; Laundal et al. 2018) further supporting this view of events.

In that case, the earlier observation of identified ‘mirroring’ ions should be reconsidered. It is unclear as to why the ion upflow/outflow would spatially limited to the poleward edge of the cusp during this flight. One could argue that these observations are actually the same phenomena, ion upflow and onset of transverse heating by the broadband wave signatures; Both populations of ions occupy similar ion energy ranges and display the same frequency range of broadband wave features. If there was ion upflow present throughout the flight, perhaps too low in energy to be observed by TRICE-II when not actively heated by ELF/VLF waves, that would support this conclusion. If indeed the earlier observation was magnetospheric precipitating ions, then understanding what factors limited this outflow spatially to the poleward side of the cusp becomes the primary interest. This answer lies in direct observation of the ‘inferred’ (red boxes) ion energization processes labeled in Fig. 3.

Dual measurements by OCHRE and TRACERS offer a unique opportunity to correlate several phenomena of low altitude ion heating and subsequent outflow. In particular, investigating the temporal and spatial variation of ion upflow, altitudinal range of the ELF/VLF occurrence region, and measuring direct ion/electron heating and scale height will be key in distinguishing between ion source populations (magnetospheric vs ambient heated ionospheric), developing an understanding of cusp outflow at low altitudes and shining light on the questions presented by TRICE-II observations.

### SQ3: Can High Resolution Measurements of the Cusp Electron Edge Describe the Temporal Variability in Newly Reconnected Field Lines?

Upon entering the cusp, TRICE-II observed the broad dispersed onset of precipitating ions. This dispersion feature, with the higher energy ions being observed first, at lower magnetic latitudes, and the lower energy ions being observed later, at higher magnetic latitudes, is a feature associated with dayside reconnection, and is much more prevalent in ion data than electron data due to the relative mass differences between the particles. Ion dispersions can display features such as steps (Lockwood et al. 2001), plateaus, and overlapping dispersions (Fuselier et al. 2022; Burkholder et al. 2024), that have been connected to temporal or spatially variability of magnetopause reconnection. Electron dispersions from dayside reconnection have not been observed before and are a missing piece in understanding the temporal variability of dayside reconnection. Particular interest lies in observing these electron dispersions within the cusp electron edge. Present in the TRICE-II data at about 8:34:30 UTC, Fig. 1, this sharp onset of precipitating solar wind electrons is defined by the open/closed field line boundary (OCB) (Lockwood et al. 2005; Øieroset et al. 2015), and is present during active dayside reconnection. The electron edge contains the most recently reconnected field lines, and observations in this region could also reveal information about dynamics of the electron diffusion region. In lieu of previous observations, simulations can provide context on what OCHRE and TRACERS could expect to observe.

**Fig. 4:**
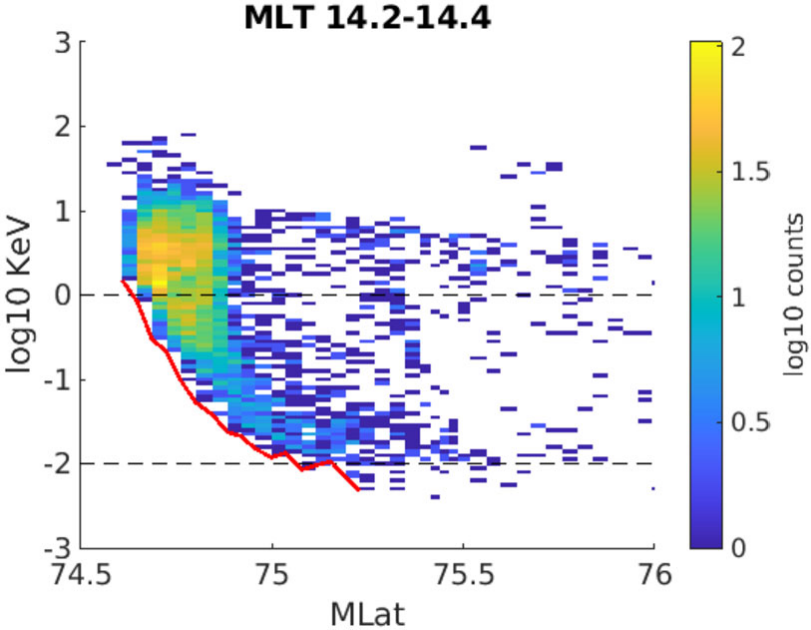
A simulated electron dispersion feature binned from 14.2-14.4 magnetic local time

To support OCHRE and TRACERS science, Grid Agnostic MHD for Extended Research Applications (Zhang et al. 2019; Sorathia et al. 2020) and Conservative Hamiltonian Integrator for Magnetospheric Particles (Sorathia et al. 2018) particle simulations are being employed to explore electron dispersion features and estimate what might be observable with OCHRE and TRACERS. Simulations estimate the $\Delta $t between ∼1 keV and ∼0.01 keV energy particles, marked by black lines in Fig. 4, traveling from the point of reconnection to the edge of the simulation boundary, at 6 $R_{E}$, to be 41 seconds. This corresponds to a spatial distance of ∼2000 km covered by this dispersion, due to field line convection (marked by the red line in Fig. 4). This is ∼4 times the current grid size of the simulations and supports the belief that current simulations can capture these electron dispersion features well. Projecting this dispersion distance down field lines to OCHRE and TRACERS altitude, the spatial scale becomes ∼225 km and ∼165 km respectively. The spatial resolution of the OCHRE electron instrument is 50 meters, vs 350 meters for the TRACERS counterpart. This means that OCHRE would achieve an order of magnitude more observations, ∼4000 samples vs ∼450 samples for TRACERS, within this electron dispersion.

Additional particle dispersion below 6 $R_{E}$ is not yet considered in these simulations. Changing field line convection speed with altitude and convection direction will both affect the orientation and size of these dispersions. Considering the TRICE-II observations as an example, as it launched into B_*y*_ dominant IMF with a slightly eastward from north trajectory, the particle dispersions would have been visible primarily across magnetic local time and the rocket would not have hit the cusp directly perpendicular; both of these factors would affect the spatial size of possible electron dispersions observed. In the above analysis, utilizing southward dominant IMF, the electron dispersion sizes mapped down to 1200 km would lie primarily along magnetic latitude, and along OCHRE’s northward trajectory.

The ideal case for electron dispersions, presented above, illustrates the advantage OCHRE has over TRACERS in observing these spatially narrow phenomena in the northern cusp. With an order of magnitude more observations, OCHRE also has the spatial resolution necessary for potential observations of substructure within the broader electron dispersions that may be evidence of quick, sub-second, temporal variations in the reconnection rate, such as that reported by Pritchard et al. (2023) in their survey of dayside reconnection rates (RRs). Such RR variability implies that electron steps would be a common feature within observations of these particle dispersions. OCHRE and TRACERS observations will also help to constrain the low altitude spatial variation of these dispersions to improve modeling.

## Instrumentation

The instrument suite for OCHRE was chosen to mimic the TRACERS instrumentation. By incorporating design improvements and developments made for TRACERS, OCHRE is able to fly higher quality instruments and improve our chances for success. The similarity of the instrument suites (identical or similar energy ranges, measurement cadences, and energy resolutions) also allows for easy comparison of the data between the spacecraft. The OCHRE payload will include two electrostatic analyzers, Cusp Electron Dynamics Instrument (CuEDI) (cf. Halekas et al. 2025) and Student Analyzer for Cusp Ions (SACI) (cf. Fuselier et al. 2025), a Fields suite (Fields) featuring a 2-D Electric Field Instrument (EFI) (cf. Bonnell et al. 2025) and a commercial fluxgate magnetometer (Mag), two Miniature Tesseract fluxgate magnetometer technical demonstrations (MiniT) (cf. Miles et al. 2025b), a Search Coil Magnetometer (SCM) (cf. Hospodarsky et al. 2025), and two Langmuir Probes (LPs).

A rocket suite will also be supplied by Wallops Flight Facility. This suite includes an Attitude Control System (ACS) for use in flight to maintain alignment to the magnetic field (particularly during the science region of interest). As well as a GPS and accelerometers that provide position, altitude, velocity, acceleration measurements, and body roll rates.

### Cusp Electron Dynamics Instrument (CuEDI)

**Fig. 5:**
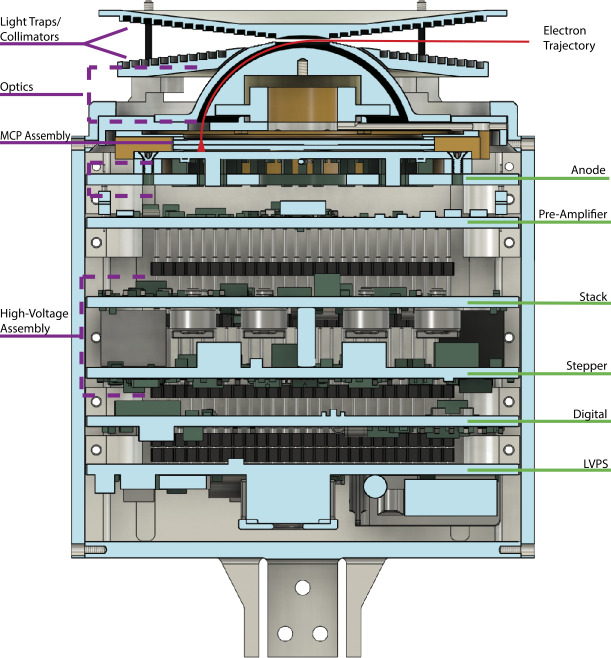
Cutaway view of CuEDI. A radial electric field guides electrons of appropriate energy through the optics to an MCP assembly, which then deposits a measurable charge on detection anodes. Adapted from Halekas et al. (2025)

Electron differential energy flux and pitch angle measurements will be taken using a boom-mounted top-hat electrostatic analyzer (ESA), CuEDI. This instrument features three sections: an optical assembly, anode assembly, and supporting electronics, see Fig. 5 for details.

The optical assembly is comprised of two concentric nested hemispheres. The larger hemisphere features an entrance hole surrounded by an upper and lower collimating light trap, completing the classic ‘top hat’ look. A positive voltage is applied to the inner hemisphere, creating a radial electric field to divert electrons. A sweep of 50 voltages will be applied to the inner hemisphere, with each step lasting 1 ms, such that a complete energy distribution will be measured over 50 ms. The voltage range determined is to measure electrons from ∼10 eV to ∼20 keV, with a < 20% $\Delta $E/E. After being redirected by 90^∘^ from the collimators, the electrons strike the chevron-pair microchannel plates (MCP).

The MCP magnifies the signal by a factor of ∼10^6^, and each pulse is deposited on one of 21 metallic anodes. Each anode covers 10^∘^ of angular resolution, totaling a 210^∘^ azimuthal view with a 7^∘^ elevated view (determined by the geometry of the collimators). If the rocket is aligned with the magnetic field, these 210^∘^ will cover pitch angles ranging from −10^∘^ to 190^∘^, ensuring that CUEDI produces a full energy and pitch angle distribution every sweep. Each pulse of electrons is fed to a pre-amplifier circuit, checked against a count threshold voltage, and passed to the digital board FPGA.

The remaining electronics consist of the Low Voltage Power Supply (LVPS), digital, stepper, and stack boards. The LVPS mediates rocket power for CUEDI, while the digital board mediates telemetry to the rocket, digitizes science and housekeeping data, and maintains the count threshold voltage and voltage sweep values. The stepper and stack boards generate the high voltage steps swept on the inner hemisphere and hold the voltage across the MCP respectively.

**Fig. 6:**
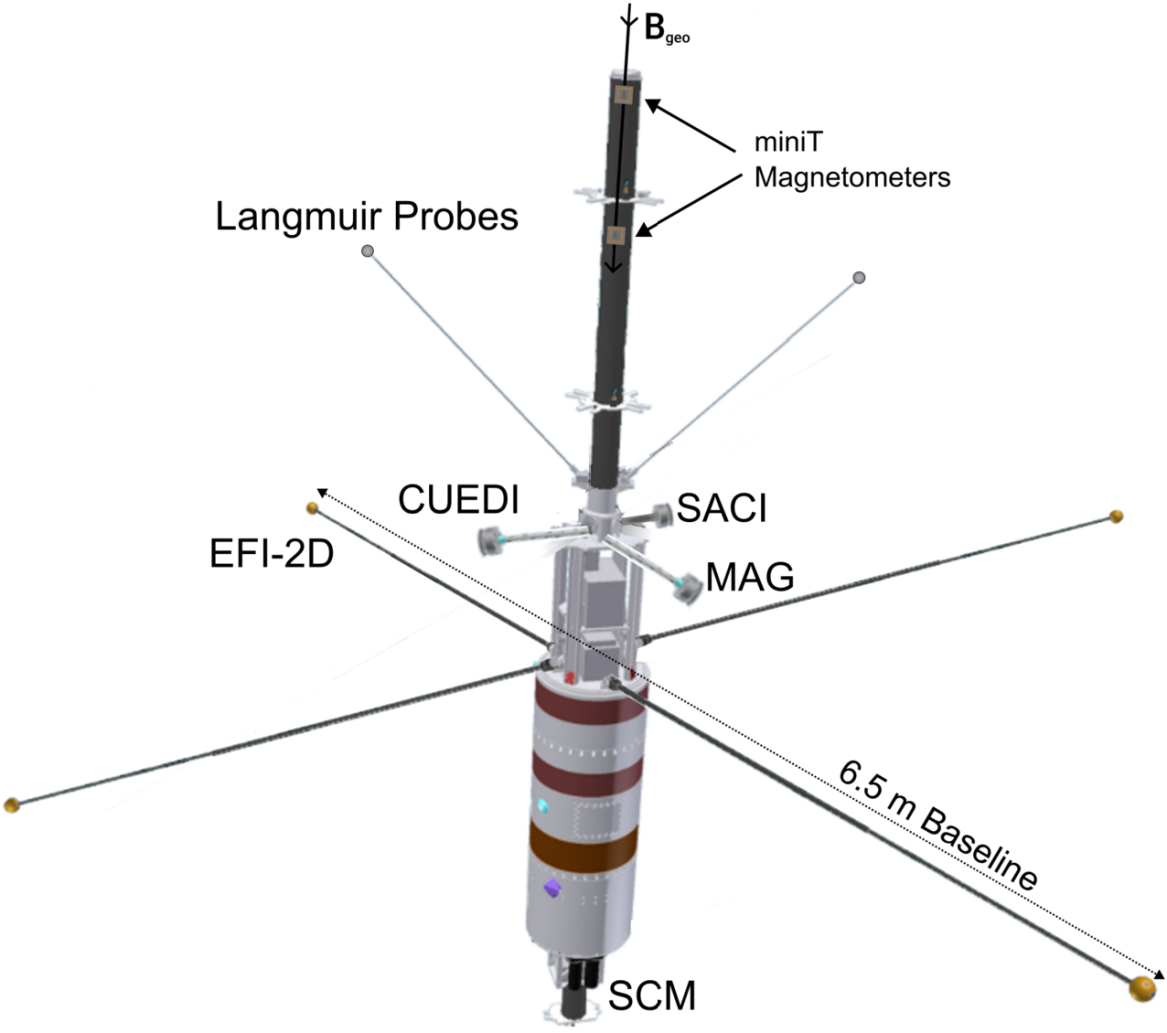
Instrumentation mounting and location diagram aboard OCHRE

The precursor of CuEDI, EEPAA, flew on board the TRICE-II and ACES-II sounding rockets. The Analyzer for Cusp Electrons (ACE), the TRACERS counterpart to CuEDI is also descendant from EEPAA. CuEDI draws heavily from the electronics and board design improvements made for ACE, for details see Halekas et al. (2025), while maintaining the optical assembly from EEPAA. ACE also features a panel-mount design directly to the TRACERS body, while CuEDI will be mounted on a flip-out boom supplied by the University of Iowa (see Fig. 6). After deployment, the boom provides an unobstructed view (30 degrees laterally across all 210 degrees polar) for the CuEDI optics.

CuEDI will be able to produce high resolution electron flux measurements that will be a key observation in all of OCHRE science. The PADs from CuEDI will allow for identification of electron beams, relating to large scale E_∥_, as well as determining energy deposition rates by electron precipitation. CuEDI also has the energy and temporal resolution necessary to make fine scale observations of the electron edge and observe any possible reconnection related electron dispersions.

### Student Analyzer for Cusp Ions (SACI)

**Fig. 7:**
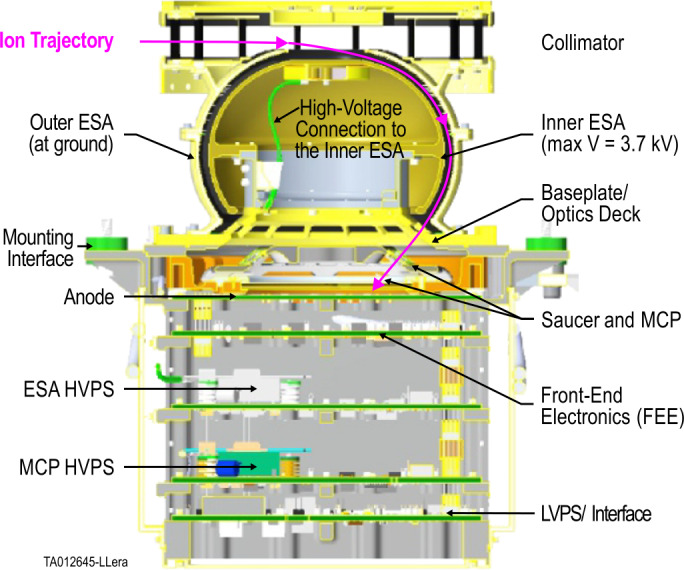
Cutaway view of SACI. The ion trajectory enters the collimator and ions with appropriate energy per charge traverses the ESA gap and strike the microchannel plate (MCP) detector. The signal is amplified by the front-end electronics (FEE) and then transmitted to be processed onboard the rocket e-box. Adapted from Fuselier et al. (2025)

The Student Analyzer for Cusp Ions (SACI) is a toroidal top-hat ESA, designed to resolve the energy-pitch angle distribution of polar cusp ions. The ‘top-hat’ ESA comprises inner and outer toroidal hemispheres 4 mm apart. The entrance collimator provides a 6^∘^ by 360^∘^ field-of-view (FOV), and a total ion redirection angle of 128^∘^. A high-voltage bias is applied to the inner toroid over 47 discrete voltage steps ranging from ∼1 V to 3.7 kV, over 312 ms. The voltage range is designed to filter an energy-to-charge (e/q) ratio from ∼8 eV to ∼20 keV (for H^+^ ions) with a $\Delta $E/E of <20%. Ions with the appropriate e/q exit the ESA and strike the MCP detector. The ion signal is then amplified and collected on 16 discrete pitch angle anodes. The ion trajectory and key instrument assembly are shown in Fig. 7.

The electronics consists of four primary boards, each with its own FPGA. The Front-End Electronics board processes the output pulses from the MCP, amplifying the signals and converting them to digital outputs. The interface and LVPS board manage the power distribution and transmits processed data to the rocket electronics. There are two High Voltage Power Supply (HVPS) boards. The first one generates the sweeping high-voltage supply to select ion energies through the ESA, and the second provides a static supply for the MCP. The energy steps are roughly logarithmic, but the lower energy bins have linear steps for a finer resolution of the lower-energy ion distributions.

SACI mostly follows the design from TRICE-II with some earlier heritage from the Hot Plasma Composition Analyzer on board the Magnetospheric Multiscale Mission satellites, and is a variant of the Analyzer for Cusp Ions (ACI) onboard TRACERS, for details see Fuselier et al. (2025). Similar to ACE, ACI features a panel mount design directly to the TRACERS body, while SACI will be mounted on a deployable mechanism, supplied by Wallops Flight Facility, that extends the collimator approximately 4 cm beyond the rocket payload deck (see Fig. 6) and aligns the FOV along the rocket body to allow for full velocity and pitch angle distributions (PADs).

SACI’s high-resolution measurements of ion velocity and PADs will allow OCHRE to observe the evolution of these distributions throughout the flight. Complimenting TRACERS, the higher altitude measurements of reconnection related features, velocity dispersions most commonly, will add valuable data for understanding the temporal and spatial variability of reconnection. Classifying velocity and PAD evolution in response to broadband wave features, observed with EFI, will help to characterize the ion heating mechanisms, and shed light on the open questions from the TRICE-II observations.

### Fields Suite

The Fields package aboard OCHRE consists of a 2-D electric field instrument (EFI), and a commercial fluxgate magnetometer (Mag). The electric field sensors are 8 cm diameter graphite-coated (DAG-213) aluminum spheres mounted on 3 m stacer booms for a spin plane EFI. The spin plane stacer booms are part of a single Quad Stacer Boom deployment system with a single release point via a NSROC-provided pyro pinpuller for all four stacer booms. The controlled release rate leads to a balanced, uniform deployment and quiet vehicle dynamics. Individual sensor potential measurements up to ±13 V will be taken at 16-bit resolution with sampling up to 2 kHz (1 kHz Nyquist). Electric field measurements will be produced using the standard double-probe technique outputting 2D DC/LF (at ∼2 ksamp/sec) and VLF measurements (at ∼ 32 ksamp/sec) of spin plane electric field (up to 1 V/m (adjustable) at ∼1 mV/m accuracy). Previous observations have shown that the majority of the low frequency electric field fluctuations are perpendicular to the background magnetic field, while parallel electric field fluctuations are present at higher frequencies and tend to be extremely weak. To close our science goals for the OCHRE mission, we are interested in low frequency electric field fluctuations associated with the BBELF signatures, and thus will not have a direct measurement of the axial electric field component. The EFI draws heritage from previous missions since the 1980s, and most recently from sounding rocket payloads ACES-II, GREECE, and TRICE-II. The EFI aboard OCHRE is consistent with the 2048 samp/s DC/LF E and V channel Region of Interest (ROI) data products from TRACERS EFI, for details see Bonnell et al. (2025).

Mag will be a commercial Billingsley TFM65-VQS fluxgate magnetometer, and will produce 3D DC/LF fluxgate magnetometer measurements with 16-bit resolution up to 2 ksamp/sec over a $\pm \mathrm{100~\mu T}$ range. The Mag will be payload mounted on a spring-loaded fold-down boom, as shown in Fig. 6, with heritage from TRICE-II, VIPER, INCAA, and ACES-2.

**Fig. 8:**
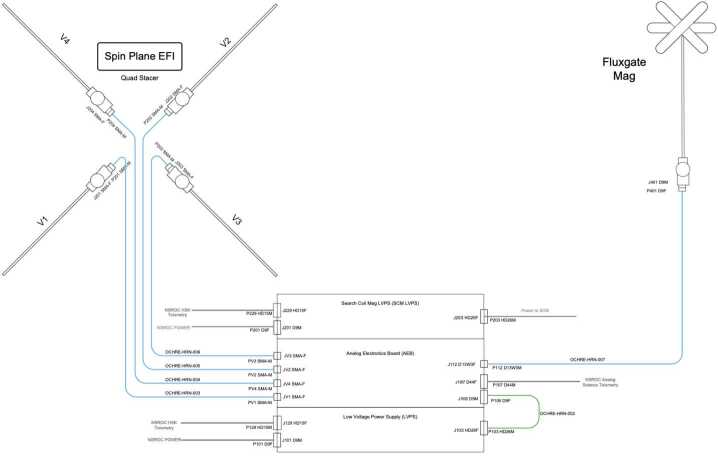
Functional block diagram of the OCHRE Fields suite showing the EFI, commercial Mag, and FEB. Courtesy of R. A. Hochman

Power and analog signal processing for both the EFI and Mag will be fed through the Fields Electronics Box (FEB) providing a single interface for both instruments with a serial digital interface to NSROC telemetry. Instrument housekeeping data such as temperatures, currents, and voltages will be outputted through this interface. This configuration has recently flown on a variety of sounding rocket missions (GREECE, TRICE-II, VIPER, INCAA, and ACES-II) and shares heritage with satellite projects such as Polar, THEMIS, and the Van Allen Probes. The FEB will also house the power board for the SCM as described in Sect. 3.5. A functional block diagram showing an overview of the Fields suite is shown in Fig. 8.

The Fields package will produce high resolution measurements of floating potentials, electric and background magnetic fields. Electric fields and interferometry transverse to the background field will allow for Poynting flux measurements, and $\mathrm{E }\times \mathrm{B}$ drift analysis to look at Alfvénic fluctuations and reconnection signatures. Poynting flux measurements will be produced using the UCB Mag and EFI measurements. DC/ULF measurements of Poynting flux can be correlated with ion upflow signatures and allow the study of local ELF/VLF waves to correlate with electron reconnection signatures. The frequency range of this suite will allow characterization of broadband wave signatures using spectral and cross-spectral measurements. In conjunction with SACI measurements, estimates for ion scattering and heating rates will also be possible.

### Miniature Tesseract (MiniT)

**Fig. 9:**
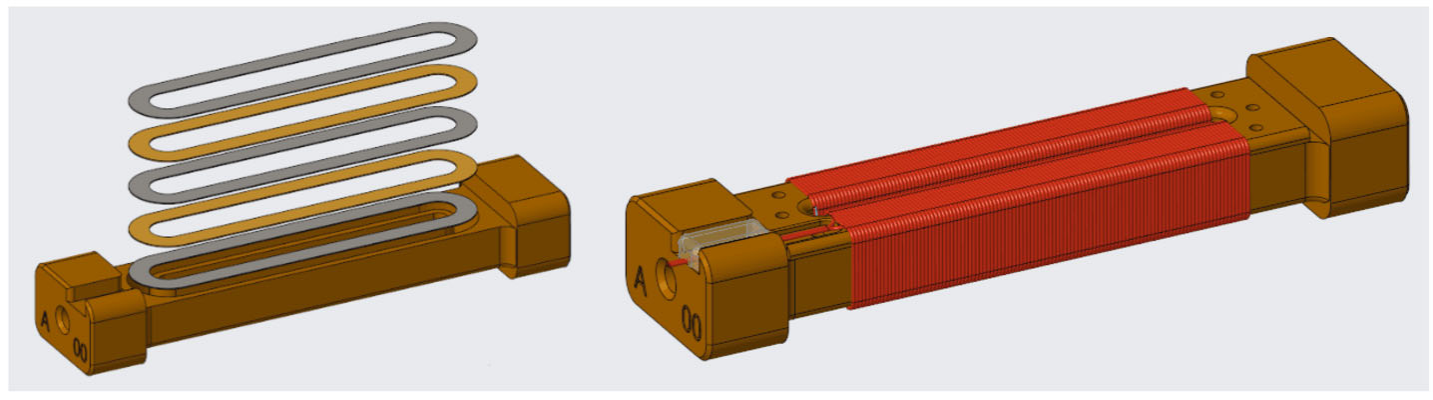
Detailed view of the racetrack core shape with the copper sense windings. Adapted from Greene et al. (2022)

Fluxgate magnetometers, common workhorses of space physics, measure the static and low frequency magnetic field through a process that involves pumping ferromagnetic cores into magnetic saturation and measuring the asymmetric modulation response due to the local magnetic field. Two of the University of Iowa Mini-Tesseract (MiniT) fluxgate magnetometers will be onboard the OCHRE mission. The sensors’ ferromagnetic cores are manufactured at the University of Iowa, applying the “racetrack” geometric design (Greene et al. 2022). This involves melting metal powders to form ingots, milling thin foil from the ingots, machining to fabricate the “racetrack” washers, Fig. 9, winding copper wires around cores by hand and testing the noise sensing of the cores. The in-house design of the cores allows for the minimization of noise from the cores and improved measurement accuracy through careful control of the entire manufacturing process. (Miles et al. 2019).

**Fig. 10:**
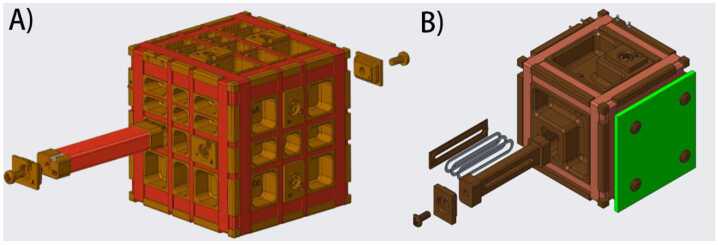
(A) The tesseract sensor flown aboard the ACES-2 sounding rocket, with the racetrack core placement and housing shown. Similar in design to MAGIC, to be flown aboard the TRACERS spacecraft. Also note the 4 coil (Merritt) feedback winding. Adapted from Greene et al. (2022). (B) 3D model of MiniTs to be flown aboard OCHRE. Note the added external electronics, the single core per axis, and the two coil feedback winding

The MiniT sensors are mounted on a fiberglass mast at the nose of the rocket to create as much distance as possible between the instruments and the AC noise and DC offsets produced by the payload and its electronics (see Fig. 6). MiniT is capable of measuring the background (DC) magnetic field with a resolution of <.05 nT and an accuracy of <10 nT and a total capable range of ± 65,536 nT at a sampling rate of 256 samp/sec. The MiniT is an updated version of the heritage design of the Tesseract fluxgate magnetometer shown in Fig. 10A. A science and technology demo under NASA Procedural requirements 7120.8A technology demonstration. The first science and technology demonstration of this sensor was the ACES-2 sounding rocket flight (Greene et al. 2024).

**Fig. 11:**
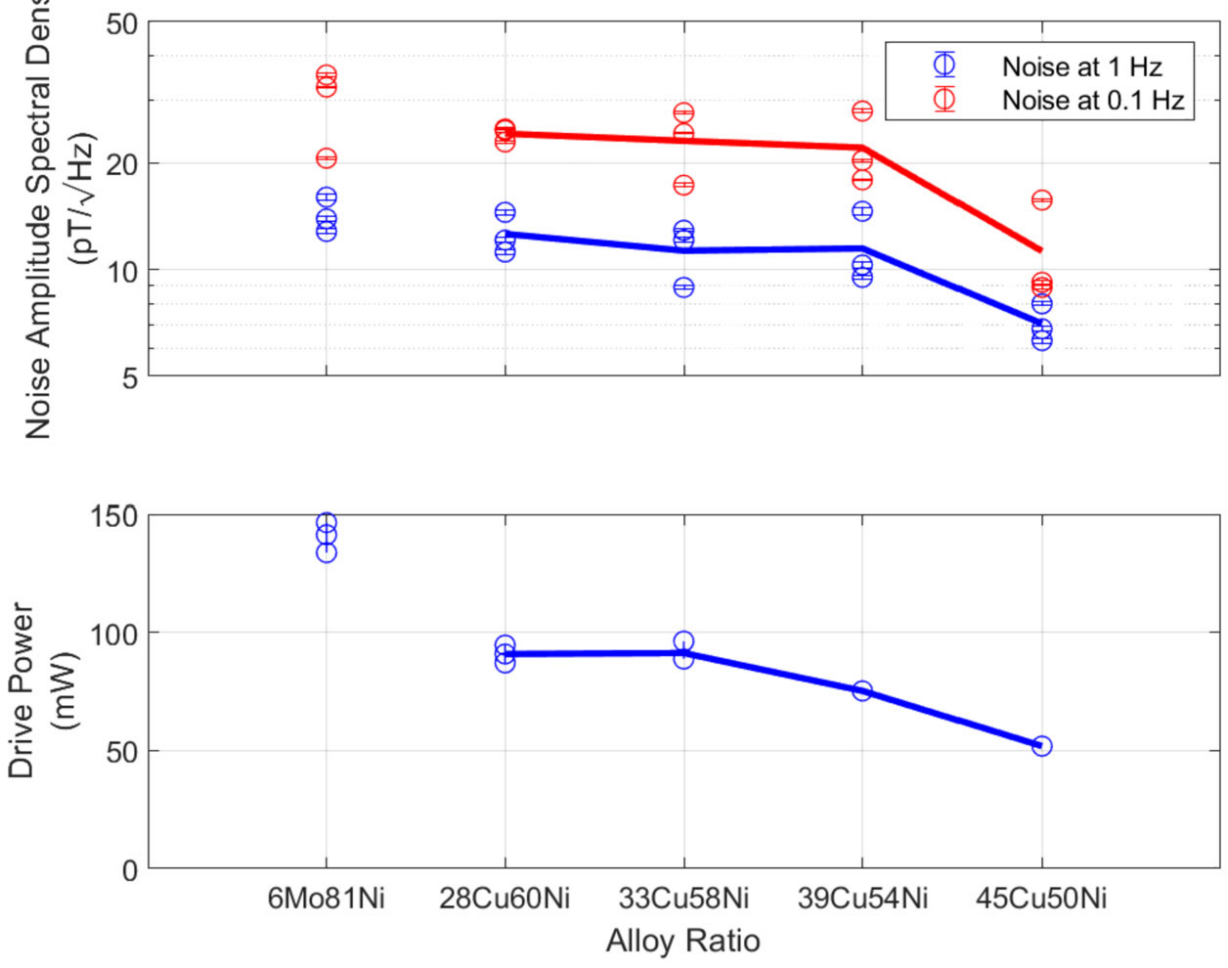
Noise and power consumption associated with different alloy ratios for racetrack geometry ring cores. Adapted from Narod and Miles (2024)

**Fig. 12:**
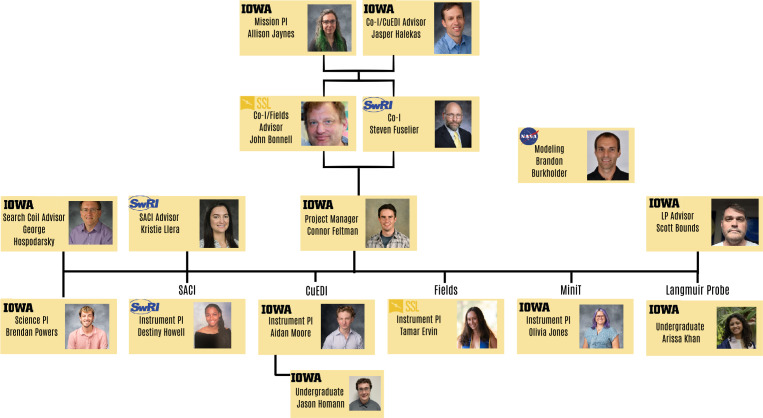
The OCHRE team organizational structure and defined roles

MAGnetometers for Innovation and Capability (MAGIC), a Tesseract magnetometer, will be flying on board TRACERS as a technical demonstration. Compared against MAGIC, for details see Miles et al. (2025b), MiniT utilizes one core per axis to MAGIC’s two. The sense and feedback windings, separate on MAGIC, are one and the same on MiniT and are now a classical Helmholtz 2 coil configuration, as compared to the 4 coil Merritt coil configuration aboard MAGIC (see Fig. 10). The permalloy for ferromagnetic cores in fluxgate magnetometers historically is a ternary of 6% molybdenum, 81% nickel, and backfilled with iron. Work in Narod and Miles (2024), highlighted a ternary of 45% copper, 50% nickel, and 5% iron that result in cores which produce less magnetic noise, see Fig. 11. One of the MiniT sensors onboard OCHRE will include three cores of this experimental ternary, while the other sensor will have the more commonly used core alloy.

MiniT is primarily a technology and science demonstration of the adjustments made from MAGIC, and the in-situ comparison between MAGIC and MiniT will be extremely useful in evaluating both instruments. OCHRE is also a preliminary comparison of the MiniT racetrack alloys compositions. MiniT will add to the scientific capabilities of the mission by measuring BBELF wave power below ∼128 Hz. Along with the Fields suite, MiniT will be used to confirm rocket alignment to the magnetic field and calibrate pitch angle measurements for our onboard particle instruments CUEDI and SACI. MiniT is also the best suited instrument for measuring the lowest frequency (DC) magnetic waves. Overlap in the higher frequency range of MiniT and the lower range of search coil magnetometer (SCM) also onboard allows for cross calibration between the two instruments. The potentially lower noise of MiniT over the Fields Mag would also provide benefit for identifying DC magnetic field perturbations related to field aligned current observations throughout the flight.

### Search Coil Magnetometer (SCM)

The SCM featured on OCHRE will measure the higher frequency magnetic field variations. Search coils are frequently used for magnetic field measurements as they have a much higher frequency roll off at a few tens of kHz as opposed to tens of Hz for traditional fluxgates. The SCM features three orthogonal 15 cm $\mu $-metal cores with dual sensor coils, and will be mounted on the bottom of the rocket payload, with two axes aligned to the EFI spin plane axis and the third aligned along the body of the rocket. All SCM electronics will be integrated into the FEB to allow for single interface from NSROC power.

The particular search coil design flying on OCHRE will be the SCM engineering model (EM) from the TRACERS satellites. Details on the SCM can be found in Hospodarsky et al. (2025). This design draws heritage from ISEE-3, Wind, Van Allen Probes and Juno satellites. Use of the EM from TRACERS leverages the depth of design improvements made for TRACERS, and will help with the ease of data comparison between the spacecrafts.

SCM will be operating at half the EFI sample rate to measure field variations up to 8 kHz (16 bit samples at 16 ksamp/sec) to measure the full spectrum of BBELF signatures present and allow for easier comparison with the Fields suite. Along with MiniT, comparison of the SCM with TRACERS will also reveal the temporal/spatial nature of BBELF wave signatures, and provide context for TRACERS to deconstruct Doppler shifted wave structures.

### Langmuir Probes

The Langmuir Probes (LPs) onboard OCHRE will be provided by the University of Iowa. One probe features a, ∼ −5 V, fixed voltage and one with a sweeping voltage, ∼ −4 - 3 V, that produce plasma density and temperature measurements at a high cadence. A variant of this Langmuir probe has flown on the HiBAR, CHARM, CHARM-II, ACES, TRICE, TRICE-II, CAPER and CAPER-II rockets. The earlier variant used a linear transimpedance stage followed by a logarithmic digitization stage. The current version flew on ACES-II and utilizes a logarithmic transimpedance stage followed by a linear digitization stage, improving measurement accuracies at lower densities. The LP electronics feature two separate circuits to support the probes, one for the fixed bias mode and the other for the swept bias mode. The fixed bias circuit also has two measurement channels, a logarithmic current measurement, and a band pass filtered signal providing a $\Delta $N/N measurement. Mechanically, the Langmuir probes consist of a 36 inch carbon fiber shaft with a one inch diameter metal probe at the end that store parallel to the rocket body during launch and deploy from a spring loaded mechanism to rest at 30^∘^ from rocket normal (see Fig. 6). The fixed LP will be operating at 1 ksamp/sec, and telemetry and power for both probes is fed through the LP electronics box. The swept LP will take one measurement every millisecond for 10 ms at every voltage, giving us a full triangular voltage sweep at the 1 ksamp/sec rate matching the fixed LP. The LPs are the only instruments with no direct counterpart onboard TRACERS, and will supplement particle measurements from CUEDI and SACI.

The fast sample plasma density measurements will be key in identifying signatures such as that postulated in the pressure cooker scenario, see Fig. 2, or density variations relating to FACs. The LPs will also be useful in broadband wave mode identification as several of the proposed candidate wave modes for BBELF waves should be visible in their effects on density structuring. Plasma temperature measurements, in coincidence with EFI wave power measurements, will help characterize the altitudinal and spatial range of BBELF wave heating.

## Conclusion

Observing Cusp High Altitude Reconnection and Electrodynamics, OCHRE, presents a unique opportunity for the education of a broad coalition of undergraduate, graduate students, postdoctoral, and early career researchers. The team structure outlined is a first of its kind mission architecture made possible by the broad support of the TRACERS science team. OCHRE is well instrumented to help answer a variety of open questions of cusp dynamics resulting from the TRICE-II observations. In concert with TRACERS, OCHRE will also look at the altitudinal and temporal evolution of reconnection related ion and electron velocity dispersions. This mission structure maximizes the educational and science output of a traditional sounding rocket mission and serves as a format for training the next generation of space researchers.

## Figures and Tables

**Fig. 13:**
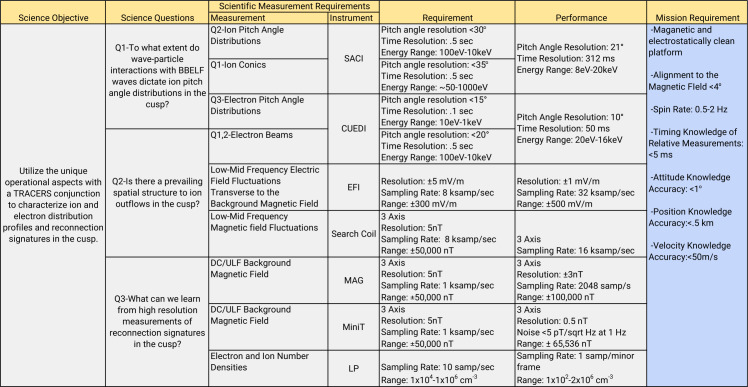
The OCHRE science traceability matrix. Defines the instrument requirements necessary for closure of the proposed science questions, and the actual instrument performances
